# Effects of temperature and photoperiod on the seasonal timing of Western honey bee colonies and an early spring flowering plant

**DOI:** 10.1002/ece3.7616

**Published:** 2021-05-07

**Authors:** Gemma N. Villagomez, Fabian Nürnberger, Fabrice Requier, Susanne Schiele, Ingolf Steffan‐Dewenter

**Affiliations:** ^1^ Department of Animal Ecology and Tropical Biology Biocenter University of Würzburg Würzburg Germany; ^2^ CNRS IRD UMR Évolution, Génomes, Comportement et Écologie Université Paris‐Saclay Gif‐sur‐Yvette France

**Keywords:** *Apis mellifera*, climate change, *Crocus sieberi*, phenology, plant–pollinator interaction, temporal mismatch

## Abstract

Temperature and photoperiod are important Zeitgebers for plants and pollinators to synchronize growth and reproduction with suitable environmental conditions and their mutualistic interaction partners. Global warming can disturb this temporal synchronization since interacting species may respond differently to new combinations of photoperiod and temperature under future climates, but experimental studies on the potential phenological responses of plants and pollinators are lacking. We simulated current and future combinations of temperature and photoperiod to assess effects on the overwintering and spring phenology of an early flowering plant species (*Crocus sieberi*) and the Western honey bee (*Apis mellifera*). We could show that increased mean temperatures in winter and early spring advanced the flowering phenology of *C. sieberi* and intensified brood rearing activity of *A. mellifera* but did not advance their brood rearing activity. Flowering phenology of *C. sieberi* also relied on photoperiod, while brood rearing activity of *A. mellifera* did not. The results confirm that increases in temperature can induce changes in phenological responses and suggest that photoperiod can also play a critical role in these responses, with currently unknown consequences for real‐world ecosystems in a warming climate.

## Graphical abstract

This study analyzed whether differences in the importance of temperature and photoperiod as Zeitgebers could lead to different phenological responses of the early‐season flowering geophyte *Crocus sieberi* and *Apis mellifera* colonies under future climates. Increased mean temperatures in winter and early spring advanced the flowering phenology of *C. sieberi* and intensified brood rearing activity of *A. mellifera* but did not advance their brood rearing activity. Flowering phenology of *C. sieberi* also relied on photoperiod, while brood rearing activity of *A. mellifera* did not. Increases in temperature can induce changes in phenological responses and photoperiod can also play a critical role in these responses.

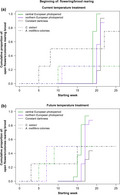

## 1 INTRODUCTION

Species that share habitats are often connected by trophic interactions which can be beneficial for one or both species. To increase the chances of co‐occurrence, interacting species need to be temporally synchronized. Many interacting species use periodical environmental factors to adapt their life cycle phenology, in particular photoperiod and temperature (Bradshaw & Holzapfel, 2007; Helm et al., 2013; Kronfeld‐Schor et al., 2017). However, climate change can dissociate the interaction between photoperiod and temperature as Zeitgebers (periodic factors in the environment capable to synchronize biological rhythms (Binder et al., 2009)). While photoperiod remains unaffected by climate change, temperature regimes are highly affected by global warming (IPCC, 2014). Consequently, current combinations of photoperiod and temperature could become unreliable Zeitgebers under future climates (Kronfeld‐Schor et al., 2017), and result in temporal mismatches between interacting species if they differ in the way how their timing relies on temperature and photoperiod (Visser et al., 2004). These changes might desynchronize the phenology of interacting species (e.g., Visser & Holleman, 2001) and cause fitness losses in one or both of them (Both et al., 2009; Hegland et al., 2009; Nürnberger et al., 2019; Parmesan, 2006; Schenk et al., 2018).

Plant–pollinator interactions maintain the reproduction of 87% of angiosperms around the world (Ollerton et al., 2011) and provide food to numerous species, including humans (Klein et al., 2007). Although this plant–pollinator synchronization is critical for both plant and pollinator fitness, it can be affected by an increase in temperature in winter and spring (e.g., Fründ et al., 2013; Kehrberger & Holzschuh, 2019) leading to fitness losses of interacting species (e.g., Schenk et al., 2018). However, it is still not well understood how the seasonal timing of this mutualistic relationship will change with global warming and with the resulting decoupling of temperature and photoperiod as Zeitgebers. For example, in models about future climate change current temperatures in central Europe are expected to occur in northern Europe in combination with much shorter day length during winter and early spring (Kovats et al., 2014). Here, we studied whether simulated future combinations of temperature and photoperiod based on the global warming scenario RCP8.5 (IPCC, 2014) affect overwintering phenology of an early spring flowering plant (*Crocus sieberi*) and the western honey bee (*Apis mellifera*).

The interaction between flowering plants and *A. mellifera* not only provides important pollination services in global crops (Kleijn et al., 2015) but also contributes to maintain ecosystems as *A. mellifera* is the most frequent floral visitor in natural habitats (Hung et al., 2018). Moreover, in temperate climate *A. mellifera* workers start foraging in early spring (Seeley, 1995) and pollinate early flowering plant species when other pollinators are scarce. It could be expected that a temporal decoupling between flowering phenology and colony development of *A. mellifera* colonies would lead to (a) decreased plant visitation rates and hence decreased plant reproduction success (Hegland et al., 2009) and (b) reduced availability of plant resources with negative consequences for honey bee diets (Hegland et al., 2009) and the colony's resource stores (Nürnberger et al., 2019).

Some of the earliest pollinator‐dependent flowering plants are perennial geophytes (“plants with renewal buds at or below the soil line” (Tribble et al., 2020)) that remain in a dormant phase during winter, grow and flower in early spring and, at the end of the flowering season, dry, and enter the dormant phase again (Dafni et al., 1981; Rees, 1966). One example of these plants is the early spring flowering plant *Crocus sieberi*. *Crocus* species are known to be visited by *A. mellifera* (Wisdom et al., 2019), and they seem to have advanced their flowering phenology due to climate warming, as is the case of *Crocus flavus* (McEwan et al., 2011). However, it is not known how future combinations of temperature and photoperiod will affect *Crocus* phenology.

Flowering plant's phenology is known to rely on photoperiod and temperature. For example, it has been shown that in plants that form dormant buds, day length can delay or advance the start of dormancy (depending on the species) (Vegis, 1964). Long‐term studies have also shown that flowering time of different plants in Europe have advanced in response to increases in temperature (Gordo & Sanz, 2005; Menzel et al., 2006). Additionally, Kehrberger and Holzschuh (2019) have shown that an increase of 0.1˚C in daily mean air temperature during late winter and early spring advanced the flowering of the perennial herb *Pulsatilla vulgaris* by 1.3 days. However, increases in temperature do not always lead to an advance in flowering, especially in plants that need cold temperatures to break their dormancy process (Asse et al., 2018; Yu et al., 2010). These differences between plant species could potentially lead to mismatches between flowering plants and pollinators if they respond differently to increases in temperature.

*Apis mellifera* colonies show seasonal changes in their foraging and brood rearing activity. During winter in temperate regions, environmental conditions prevent colonies from foraging and force them to rely on honey stored during spring and summer. To save resources, colonies refrain from brood rearing during most of this time, but already in late winter honey bees actively increase the colony temperature and start rearing brood to have a worker force available in spring when foraging activity can be resumed (Seeley, 1995). After winter, when the food storage of the honey bee colonies has diminished, the honey bees rely heavily on spring flowering plants, which themselves often rely on honey bees and other pollinators to be pollinated.

The environmental cues that trigger the seasonal changes in Western honey bee colonies are not well known. Long‐term studies indicate that *A. mellifera* workers have advanced their first appearance date in response to climate warming (Gordo & Sanz, 2005, 2006; Sparks et al., 2010), and thus, temperature might play a role in honey bee phenology. Additionally, some data indicate that the photoperiod might not be the strongest cue (e.g., it cannot induce the development of winter bees (Fluri & Bogdanov, 1987), and it is not involved in the seasonal changes of juvenile hormone (Huang & Robinson, 1995)). Interestingly, while the duration of day length alone seems to not influence the starting date of brood rearing, it can modulate the response of honey bee colonies to changes in temperature (Nürnberger et al., 2018).

Although global warming affects winter climate (IPCC, 2014), early‐season plant and solitary bee phenology (e.g., in *Osmia lignaria* (Sgolastra et al., 2011) and in *Osmia cornuta* males (Kehrberger & Holzschuh, 2019)), very little is known on how future temperature and photoperiod combinations will affect the seasonal timing of flowering plants and Western honey bees during winter and early spring. We investigated in a climate chamber experiment how early‐season phenology of the spring flowering geophyte plant Sieber's crocus (*Crocus sieberi*), and of Western honey bee (*Apis mellifera*) colonies were affected by different combinations of temperature and photoperiod regimes during winter and early spring. We monitored flowering phenology of *C. sieberi* and brood rearing activity and phenology of *A. mellifera* colonies along six combinations of temperature × photoperiod climatic scenarios.

We expected that:

- Increased winter temperatures under a global warming scenario will advance flowering phenology of plants and brood rearing activity of honey bee colonies.
- At similar temperature conditions, flowering and brood rearing activity will start earlier under photoperiod conditions of a central European location than of a northern European location with shorter day light.
- *C. sieberi* plants and *A. mellifera* colonies will differ in their respective response to new combinations of temperature and photoperiod under future global change scenarios.

​

## 2 MATERIALS AND METHODS

### 2.1 Study design

**FIGURE 1:**
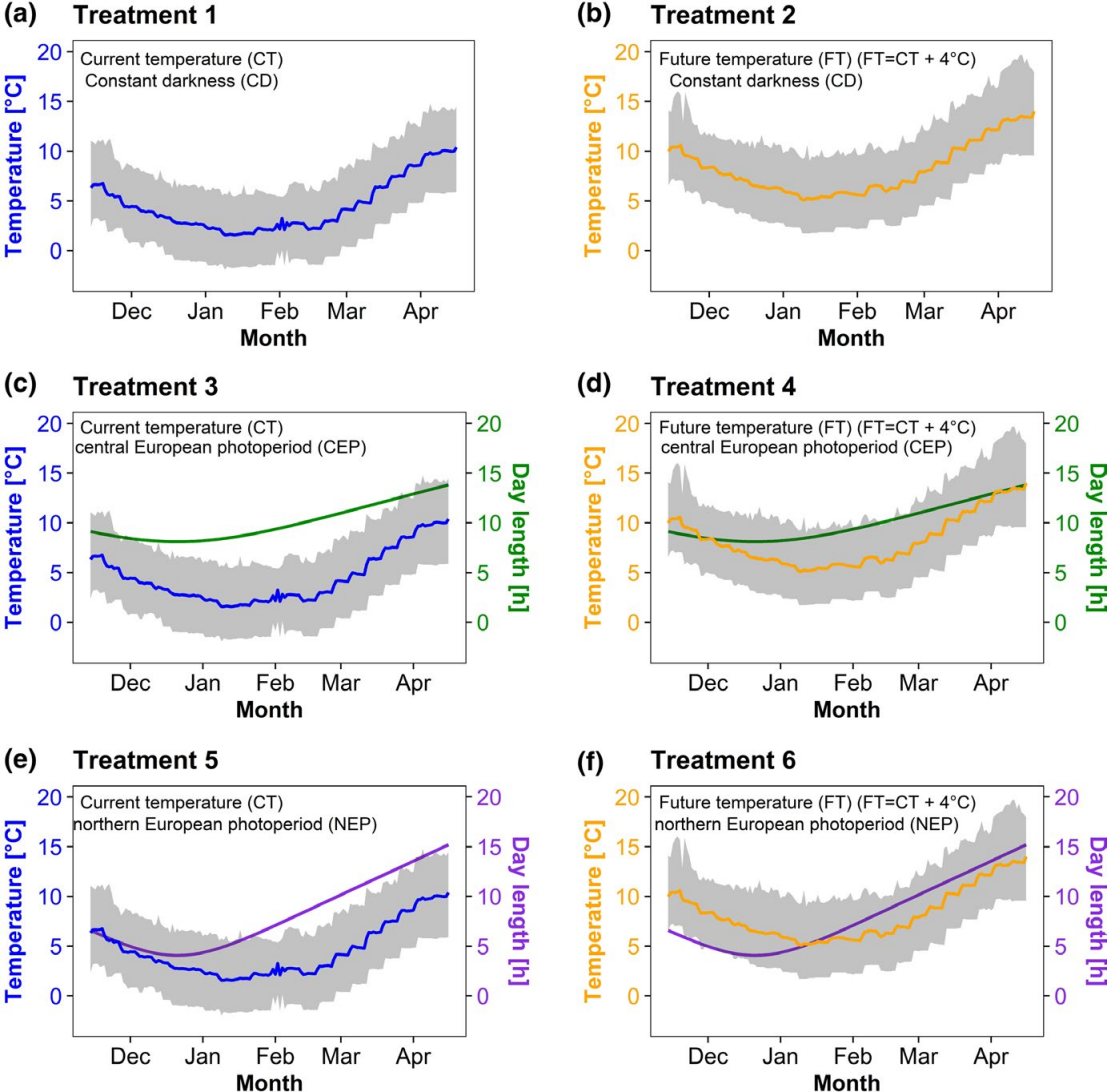
Environmental scenarios. The daily amplitude (gray area) and daily average temperature (blue and orange lines) in the two environmental chambers are shown as well as the day length of the different light regimes (green and purple lines). (a, c, e) Temperature of the current regime. (b, d, f) Temperature of the future regime. (a, b) Treatment with constant darkness. (c, d) Daily light hours in central European location (i.e., Würzburg, Germany). (e, f) Daily light hours in northern European location (i.e., Snåsa, Norway)

To study the response of a spring flowering plant and Western honey bee colonies, we established two temperature and three light regimes in environmental chambers (one temperature regime and three light regimes per chamber). One temperature regime, used as a control, simulated the average temperature during winter of a central European location (based on the weekly mean air temperature in Würzburg, Germany from 1948 to 2016), which correspond to the conditions that the species have experienced, including the increase in temperature of the past decades. The other one simulated an increase in this average temperature as predicted in global warming models. The local temperature is hereafter referred as “current temperature regime” (CT) (Figure 1a,c,e). The other temperature regime, hereafter referred as “future temperature regime” (FT) (Figure 1b,d,f), consisted of the “current temperature regime” weekly mean temperatures plus 4°C (see section: *Temperature regimes*). It was based on the global warming scenario RCP8.5, where it is expected to have a mean global temperature change of 3.7°C by the end of the 21st century (between 2081 and 2100)) (IPCC, 2014).

The light regimes were as follows: (a) constant darkness (CD) (Figure 1a,b), in order to assess the effect of temperature alone, (b) photoperiod regime of the central European location, that is, Würzburg, Germany (CEP) (Figure 1c,d), used as a control of photoperiod, and (c) a photoperiod regime representing relatively shorter day lengths at a northern European location, that is, Snåsa, Norway (NEP) (Figure 1e,f) in order to represent a scenario where the temperature and photoperiod as Zeitgebers are dissociated (see section: *Light regimes*). The photoperiod of Snåsa, Norway was chosen because its annual temperature is approximately 4°C colder than Würzburg, Germany. If an increase of temperature by 4°C occurs, the studied species could in theory shift their range northwards where they will experience the same temperatures as before but with a different photoperiod.

In each of the six unique combinations of temperature and photoperiod regimes, we established 16 crocus's bulbs (*C. sieberi* (J. Gay) ssp. *atticus*) and 4 similar sized honey bee (*A. mellifera* *carnica*) colonies. The bulbs and colonies were placed inside the environmental chambers during mid‐November 2017 (14‐11‐2017) and were kept there until mid‐spring (16‐04‐2018). During this time, their state was monitored (see the three sections: *Plant establishment and monitoring* and *Establishment of honey bee colonies*). The weeks during the experiment were numbered, with week number 1 corresponding to the week when the experiment started.

### 2.2 Temperature regimes

For calculating the weekly mean air temperature for the “current temperature regime,” we used the daily air temperature recordings from 01‐01‐1948 to 31‐12‐2016 from the German Meteorological Service (DWD) station “Würzburg” (DWD, 2017). With these data, we first obtained the mean air temperature of every day for each year. Then, with these means we calculated the mean daily air temperature from 1948–2016. Afterward, we calculated the mean air temperatures for each week as the mean air temperature of seven consecutive days starting with January 1st (the week 52 was calculated as the mean of the last nine consecutive days). For the “future temperature regime,” we added 4°C to the calculated mean weekly air temperatures.

As air temperatures are not constant during the day, we included daily temperature fluctuations in both regimes. The daily minimum temperature (mean weekly air temperature minus 3°C) occurred at 00:00 hr and the maximum temperature (mean weekly air temperature plus 3°C) at 12:00 hr. The temperature was increased hourly by 0.5°C until 12:00 hr and decreased again hourly by 0.5°C until 00:00 hr. The mean weekly temperature at the environmental chambers was always changed at the first day of the week at 00:00 hr.

### 2.3 Light regimes

**FIGURE A1:**
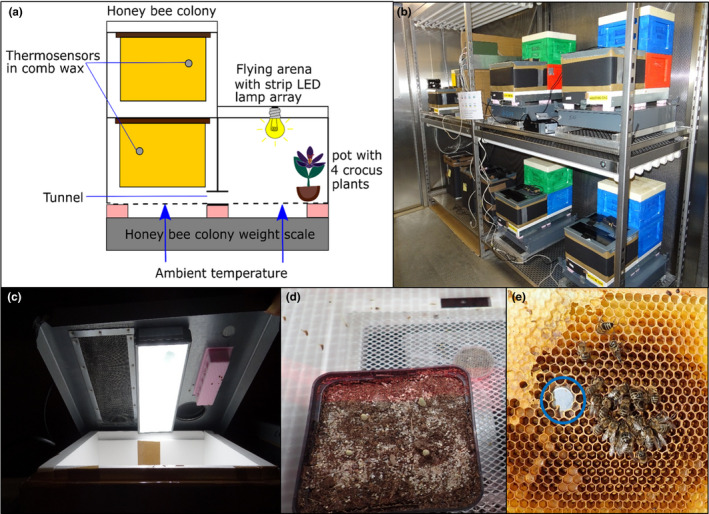
Experimental setup. (a) Scheme of the experimental setup. To measure honey bee colonies temperature, one temperature sensor was installed into each of the wax combs of the colonies. Each colony was inside two‐storied mini Plus hive boxes and placed on a beehive weight scale with remote sending data function. The bottom hive box and the flying arena had a wire mesh bottom that facilitated entry of ambient temperature. Each of colony was connected to a flying arena, which had a pot with four crocus plants and a strip LED lamp on the top. The bees were able to enter the flying arena via a short tunnel at any time (following Nürnberger et al., 2018). (b) Honey bee colonies with their flying arena on weight scales in one climate chamber. The chambers were always kept without light. (c) Into each flying arena, a strip LED lamp diffused with a sandblasted glass cover was installed to have independent light regimes for each colony. Each flying arena had a metal lid with a wire mesh that allowed to see inside the flying arena. When no observations were made, the mesh was always covered by a black high‐density polyethylene sheet. (d) Four bulbs of *Crocus sieberi* were placed inside a plastic pot, approximately 2 cm away from each other. (e) Example of a thermosensor in a comb wax. Photo credits: (b, d, e) Susanne Schiele and (c) Sandra Kehrberger

For establishing the three light regimes within the same chamber and hence independent of temperature regime, we had empty hive boxes (hereafter called “flying arena”) with its own light source: strip LED lamps diffused with a sandblasted glass cover (Appendix, Figure A1). The strip LEDs had a color temperature of 6,500 K (± 100 K) with cold white fluorescent paint and a luminous intensity of ca. 2,000 Lux (± 100 Lux). Each of these flying arenas was connected to one honey bee colony and had a pot with four crocus bulbs. The bees were able to enter the flying arena via a metal tunnel at any time, and through this tunnel at the hive entrance and the flying arena, the bees could perceive the environmental light (following Nürnberger et al., 2018) (tunnel size: 9 cm long (4.5 cm long in the flying arena side and 4.5 cm long in the hive box side) × 5.2 cm width). Each flying arena was covered with black carton, so their light could not reach the others. The flying arenas of the colonies that were kept under constant darkness were also covered to shield them from external light sources (Appendix, Figure A1).

In the light regimes central European photoperiod (CEP) and northern European photoperiod (NEP), the lamps were computer controlled and automatically turned on and off according to the local times for sunrise and sunset of Würzburg, Germany (49°48′0″ N, 9°55′48″ E) and Snåsa, Norway (64°15′0″ N, 12°23′0″ E), respectively. The NOAA Solar Calculator (NOAA, n.d.) was used to calculate the times of sunrise and sunset at the respective locations. The dawn and dusk were also simulated by gradually increasing or decreasing light intensity for 1 hr before the lights were completely on or off, respectively (software written by Dr. Conrad Wild).

### 2.4 Plant establishment and monitoring

We used bulbs of Sieber's crocuses from Albert Treppens & Co Samen GmbH. They were placed in plastic pots (9 × 9 × 9.5 cm) approximately 2 cm away from each other (4 bulbs per pot) (Appendix, Figure A1). The substrate used was a mixture of 60% soil (seed compost with 1.5 kg/m^3^ nutrient salts (Pikiererde CLP Einheitserde®)) and 40% sand (Filtersand 0.71–1.25 mm (Plantiflor®)). Three weeks after planting the bulbs, the pots were placed in the environmental chambers. The plants were watered once or twice a week depending on soil humidity.

To determine flowering phenology, plants were regularly checked and the number of weeks until flowers opened was recorded for each plant. Additionally, every week the size of the plants was measured (from the soil to the largest leaf) till the leaves started drying out. When there were two shoots coming from the same bulb, both shoots were measured, but for statistical analysis only the longest one was considered.

### 2.5 Establishment of honey bee colonies

For the experiment, we used 18 honey bee colonies headed by sister queens from the Bavarian State Research Centre for Viticulture and Horticulture (LWG) and six artificial honey bee swarms. Each of these artificial swarms consisted of 500–600 g of workers and a young mated queen (sister queens reared by Alois Kroiß). The workers were mixed from five already established honey bee colonies from the apiary of the University of Würzburg. Each colony was placed inside two‐storied mini Plus hive boxes. The colonies were treated against varroa mites, and their respective infection levels were monitored.

In order to confirm that the experiment was set up with similar sized colonies, we estimated the number of workers, capped brood area, open brood area, and area of stored honey using the Liebefelder method (Imdorf et al., 1987) before the start of the experiment. Afterward, in November 2017 we randomly allocated the colonies coming from the LWG to each treatment, while one random artificial swarm colony was allocated to each unique treatment combination.

### 2.6 Monitoring of honey bee colonies

Comb temperature in each colony was tracked using 12 thermosensors per colony following Nürnberger et al. (2018). One temperature sensor (Thermochron® iButton® device (DS1921G) 0.5°C resolution) was installed into the wax of each comb of the colonies (Appendix, Figure A1). Temperatures were automatically recorded every 3 hr. The maximum, minimum, and average temperature were determined for each day and iButton. Then, the iButton that recorded the maximum temperature for each day and colony was selected. If two iButtons within one colony recorded the same value on a given day, only one value was kept (that of the first iButton in the list). This method allowed us to identify brood rearing activity in a minimally invasive way by tracking daily temperature variation with in the winter cluster (Nürnberger et al., 2018).

**FIGURE A2:**
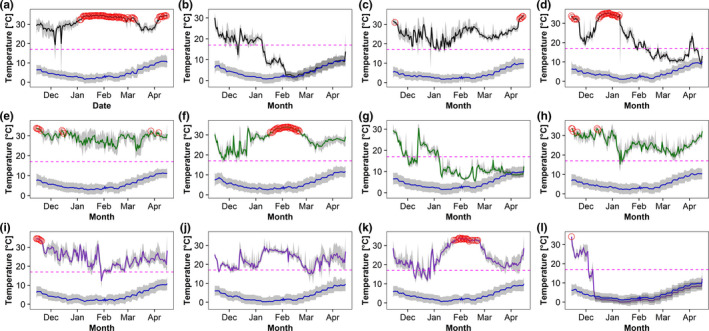
Days when *Apis mellifera* colonies reared brood under the current temperature regime. (a–d) Colonies under constant darkness (CD). (e–h) Colonies under central European photoperiod (CEP). (i–l) Colonies under northern European photoperiod (NEP). Blue lines depict the daily average temperature in the environmental chamber. Additionally, the average temperature measured at the warmest comb each day is shown. Black lines: colonies under CD, green lines: colonies under CEP, purple lines: colonies under NEP. Gray areas represent the daily temperature amplitude in both environmental chamber and comb temperatures. Red circles mark days during which colonies were inferred to rear brood (the comb temperature was >= 32°C and the amplitude <= 2°C). Pink dashed lines depict 17°C, when the temperature inside the colony was below this threshold and never recovered, it was considered that the colonies either died or were too weak

**FIGURE A3:**
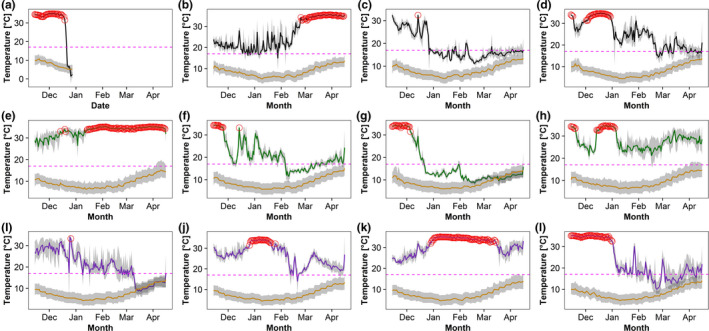
Days when *Apis mellifera* colonies reared brood under the future temperature regime. (a–d) colonies under constant darkness (CD). (e–h) colonies under central European photoperiod (CEP). (i–l) colonies under northern European photoperiod (NEP). Orange lines depict the daily average temperature in the environmental chamber. Additionally, average temperature measured at the warmest comb each day is shown: in black (colonies under CD), green (colonies under CEP), and purple (colonies under NEP). Gray area represents the daily temperature amplitude in both environmental chamber and comb temperatures. Red circles show the days when the colonies were inferred to rear brood (the comb temperature was >= 32°C and the amplitude <= 2°C). Pink dashed lines depict 17°C, when the temperature inside the colony was below this threshold and never recovered, it was considered that the colonies either died or were too weak

Honey bee brood requires a brood nest temperature between 32°C (Becher et al., 2009) and 37°C (Fahrenholz et al., 1989), and daily variations of brood nest temperature remain between 1°C (Seeley, 1995) and 2°C (Fahrenholz et al., 1989). Hence, colonies were considered to rear brood on days when the maximum temperature was equal or above 32°C and the temperature amplitude was equal or less than 2°C (e.g., see Appendix, Figures A2 and A3). It was considered that a colony either died or was too weak when the temperatures inside the colony decreased to 17°C or lower and did not recover (e.g., see Appendix, Figure A3g).

To determine the weight change of the colonies over time, we placed each colony on a beehive weight scale with remote sending data function for the complete duration of the experiment (Appendix, Figure A1). The scales were built by TrachtNet Deutschland with a data logger from Hoffmann Messtechnik GmbH and a weight scale CAPAZ^®^ GSM 200. In December 2017, the colonies were treated against varroa mite infestation with a solution of oxalic acid dihydrate 3.5% (m/V) ad us. vet. (Serumwerk Bernburg AG) in water and sucrose (60.03%). At the 04‐04‐18, a solution of sugar–water (50% Apiinvert® and 50% water) was provided to the colonies. The colonies' weight change from this day on was not included in the statistical analysis.

### 2.7 Data analysis

All statistical analyses were performed with the software R version 4.0.2 (R Core Team, 2020).

To determine whether there was a difference in the time when the flowers opened among the different treatments, a survival analysis (mixed effects Cox proportional hazard model) was performed (*coxme* R‐package*;* Therneau, 2018). For this analysis, the week when each flower opened was marked as “1.” In the case, a flower did not open till the plant dried, its information about flowering phenology was incomplete, and therefore, labeled as “0.” The explanatory variables were the temperature regime (current, future), the photoperiod regime (constant darkness, central European, northern European), and the interaction between photoperiod and temperature. Flying arena identity was included as random factor. The *p*‐values of the explanatory variables were computed with analysis of deviance based on chi‐square likelihood ratio tests (*stats* R‐package).

To compare the proportion of open flowers between the different treatments, a generalized linear model with a binomial distribution was used (*stats* R‐package). The explanatory variables were the temperature regime (current, future), the photoperiod regime (central European, northern European, constant darkness), and the interaction between photoperiod and temperature. As before, the *p*‐values were computed with analysis of deviance based on chi‐square likelihood ratio tests.

For the comparison of the final size of the plants, a linear mixed effects model was used (*lme4* R‐package; Bates et al., 2015). The explanatory variables were temperature and photoperiod regime (central European and northern European). We excluded the constant darkness regime because the plants growing in this regime suffered etiolation, which caused the plants to grow longer. This response could mask the effect of other treatments on plant growth. Flying arena identity was included as random factor. The *p*‐values of the explanatory variables were computed with type III analysis of variance and Satterthwaite's method (*stats* R‐package).

To compare the different treatment combinations in the previous analyses, contrasts of estimated marginal means (adjustment method: Tukey) were computed (*emmeans* R‐package; Lenth, 2018).

To explore if the colonies differed in the day when they started to rear brood for the first time, a survival analysis with Cox proportional hazards regression model was performed. The explanatory variables were the temperature regime (current, future), the photoperiod regime (constant darkness, central European, northern European), and their interaction. The origin of the colonies (artificial swarm or not), and if they had brood at the beginning of the experiment were also included as explanatory variables. The day when each colony started to rear brood was marked as “1.” Colonies that died or were considered to be too weak during the experiment were marked as “0” the day when the temperatures inside the colony decreased to 17°C or lower and did not recover. Colonies in which no brood rearing activity were detected, but which were alive at the end of the experiment (e.g., see Appendix, Figure A2j) were marked as “0” the day the experiment ended. We only considered the start of brood rearing activity when required temperature conditions lasted at least 3 days. To calculate *p*‐values for the explanatory variables, analysis of deviance based on chi‐square likelihood ratio tests was calculated (*stats* R‐package).

In addition, we explored if temperature and photoperiod treatments affected the likelihood of all the colonies to rear brood during the experimental time. For this, the proportion of brood rearing days compared to days without brood rearing activity was examined using a generalized linear model with a quasibinomial distribution to account for the overdispersion (*stats* R‐package). The explanatory variables were the same as above. We considered only the days before the colonies either died or when the temperatures inside them decreased to 17°C or lower. To calculate *p*‐values, analysis of deviance based on *F* test was calculated (*stats* R‐package).

We also investigated if the colonies started rearing brood before or after the flowering of the plants. For this, we performed two survival analyses with mixed effects Cox model. One model was to see the difference between the colonies and plants under the current temperature regime and the other model to see the difference under the future temperature regime. In the models, we explored if the starting week of brood rearing activity and the starting flowering week depended on the species (*C. sieberi*, *A. mellifera*), the photoperiod regime (constant darkness, central European, northern European) and an interaction between photoperiod and species. The random factor was the origin of the plants and the colonies (flying arena and artificial swarm or not, respectively).

To compare the different treatment combinations in the previous analyses, contrasts of estimated marginal means (adjustment method: Tukey) were computed (*emmeans* R‐package; Lenth, 2018).

Finally, we explored if the brood rearing status and its interaction with temperature affected the mean weight change of the colonies. Therefore, a linear model (*stats* R‐package) was implemented. The response factor was the natural logarithm of the absolute value of the mean weight change (in order to have normal distributed data). Explanatory factors were brood status (rearing or not rearing brood), temperature, their interaction, and the origin of colonies. To calculate *p*‐values for the explanatory variables, analysis of variance was used.

For all models, a significance level (*α*) of 0.05 was considered. Normal distribution and homogeneity of variance of model residuals were visually confirmed for the models.

## 3 RESULTS

### 3.1 Flowering phenology and growth of plants

**FIGURE 2:**
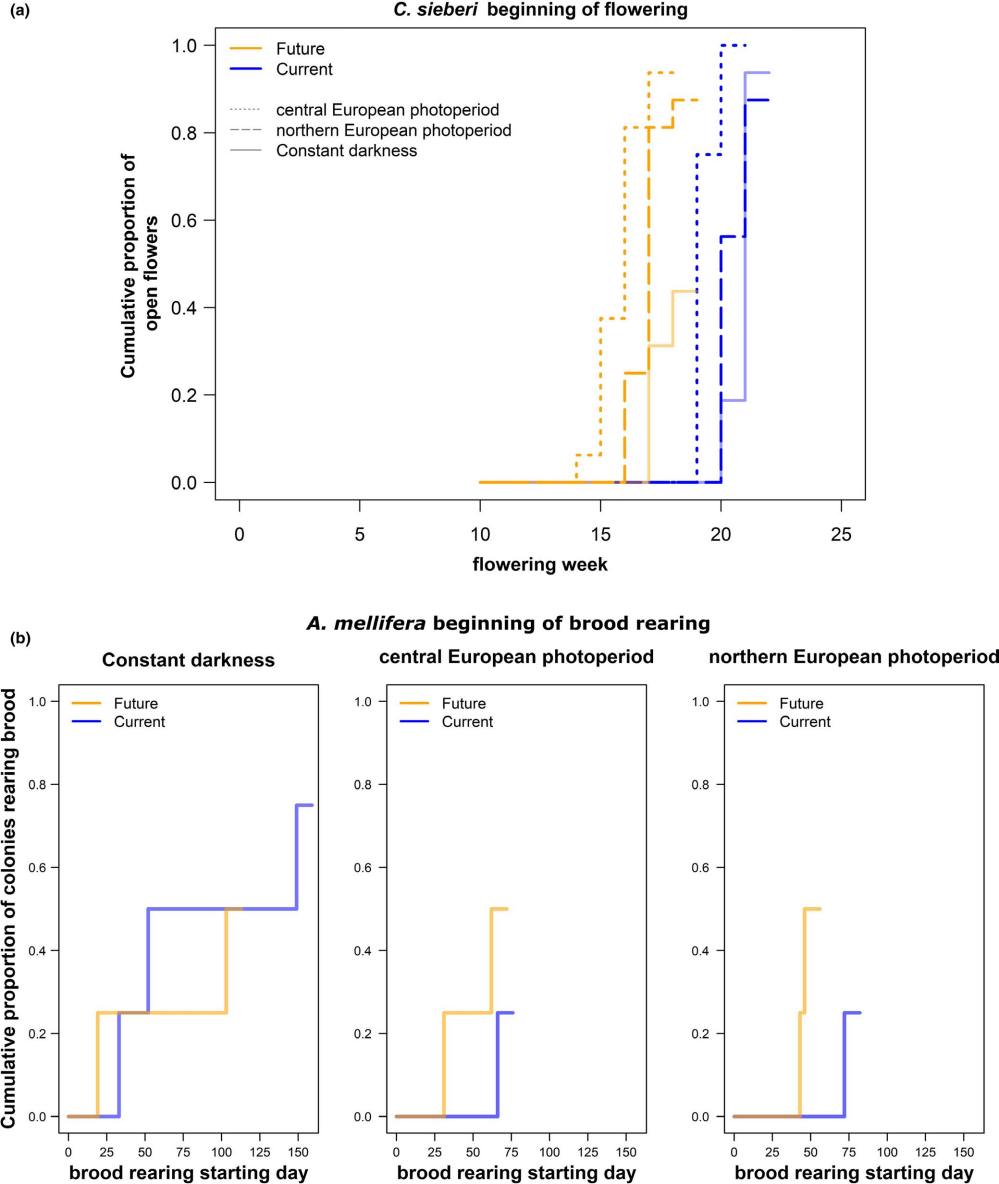
Timing of Flowering and brood rearing activity. (a) Cumulative proportion of open flowers of *Crocus sieberi* depending on the week of the experiment in the different temperature and photoperiod regimes. (b) Cumulative proportion of *Apis mellifera* colonies rearing brood depending on the day of the experiment in the different regimes. Total number of open flowers in the current temperature regime: *n* = 16 central European photoperiod, *n* = 14 northern European photoperiod, and *n* = 15 constant darkness. Total number of open flowers in the future temperature regime: *n* = 7 constant darkness, *n* = 15 central European photoperiod, *n* = 14 northern European photoperiod. Colonies with clear beginning of brood rearing in the current temperature regime *n* = 3 constant darkness, *n* = 1 in central and *n* = 1 northern European photoperiod. In the future temperature regime, *n* = 2 in each of the three photoperiod regimes

The time when *Crocus sieberi* started flowering was significantly influenced by both, temperature and photoperiod (Table 1), but only marginally influenced by an interaction between these factors (Table 1). The crocuses in the climate change scenario with increased temperatures (future regime) flowered earlier than crocuses in the current temperature regime (Figure 2a). In addition, the crocuses under the photoperiod of the central European location (CEP) flowered significantly earlier than crocuses under the treatments in constant darkness (CD) or the northern European photoperiod (NEP) in the current and future temperature regime (Appendix Table A1, Figure 2a). The crocuses under constant darkness and northern European location in the current and future temperature regime flowered approximately during the same weeks (Appendix Table A1, Figure 2a).

**TABLE 1:** Results of mixed effects Cox model and Cox model performed to test the effects of temperature and photoperiod treatments on the flowering timing of *C. sieberi* and on brood rearing activity timing of *A. mellifera,* respectively

Predictors	*C. sieberi* start flowering week			*A. mellifera* start brood rearing day		
	*χ* ^2^	*df*	*p* (>Chi)	*χ* ^2^	*df*	*p* (>Chi)
Temperature	41.4	1	**<.0001**	1.8	1	.18
Photoperiod	21.5	2	**<.0001**	1.8	2	.40
Temperature × Photoperiod	4.8	2	.09	3.6	2	.16
Origin	NA	NA	NA	4.1	1	.**04**
Brood beginning	NA	NA	NA	4.3	1	.**04**
Random effect (flying arena)						
Variance	0.36			NA		
Standard deviation	0.67			NA		

Note

Bold values indicate significant differences between treatments (*p* < .05) according to analysis of deviance based on chi‐square likelihood ratio tests.

The proportion of plants that flowered during the experiment was different between treatments (temperature treatment: *df* = 22, *p* = .009; photoperiod treatment: *df* = 23, *p* = .004; interaction: *d* = 18, *p* = .12). We observed a smaller proportion of opened flowers (mean proportion 0.44, standard deviation 0.24) in the treatment “future temperature – constant darkness” than in other treatments. In the current regime, the mean proportion and standard deviation of open flowers in constant darkness were 0.94 ± 0.13, in northern European photoperiod was 0.88 ± 0.14 and in central European photoperiod all plants flowered. In the future temperature regime, the mean proportion of open flowers was 0.88 ± 0.14 in the northern European photoperiod and 0.94 ± 0.23 in the central European photoperiod.

**FIGURE 3:**
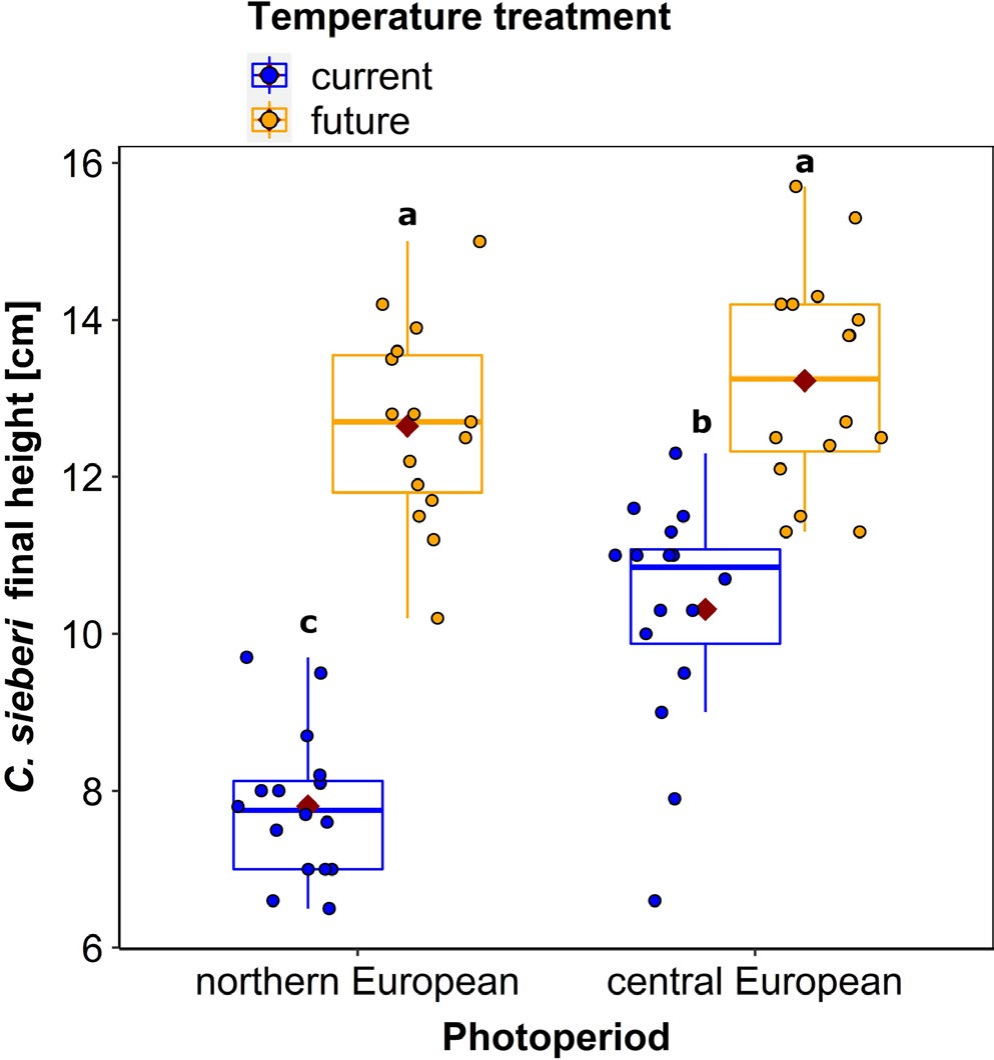
Final height of *Crocus sieberi* plants under the different temperature and photoperiod regimes. Box plots display the median (thick bar), lower and upper quartile (boxes), and minimum and maximum values (whiskers) of the data set. The red diamonds represent the mean value of the data set. Different letters indicate significant differences between treatments (*p* <.05) according to contrasts of estimated marginal means. *n* = 16 per temperature‐photoperiod treatment, except for future‐northern European photoperiod where *n* = 15

The final height of *C. sieberi* was influenced not only by temperature and photoperiod but also by their interaction (Table 2). The crocuses growing under the future temperature regime (FT) were significantly longer than the ones under the current temperature regime (CT) (Appendix Table A1, Figure 3). Additionally, the crocuses exposed to the central European photoperiod were significantly longer than the ones exposed to the northern European photoperiod under the current temperature regime. Interestingly, under the future temperature regime there were no significant differences between these two treatments (Appendix Table A1, Figure 3).

**TABLE 2:** Results of linear mixed effects model performed to test the effects of temperature and photoperiod treatments on final height of *C. sieberi*

Predictors	*F* value	*p* (>*F*)
Temperature	*F*_1,16_ = 85.8	**<.0001**
Photoperiod	*F*_1,16_ = 13.9	.**002**
Temperature × Photoperiod	*F*_1,16_ = 5.2	.**037**
Random effect (flying arena)		
*σ* ^2^	1.1	
*τ*_00_	0.4	
ICC	0.3	
*N*	16	
Observations	63	
Marginal *R* ^2^/Conditional *R* ^2^	0.75/0.82	

Note

Bold values indicate significant differences between treatments (*p* < .05) according to analysis of variance.

### 3.2 Brood rearing activity of honey bee colonies

The starting day of brood rearing varied among the colonies without a clear pattern that could be explained by the treatments (Table 1, Figure 2b). There was only a significant effect by the origin of the colonies and the brood status at the beginning of the experiment (Table 1). The colonies which did not originate from artificial swarms and the colonies that did not have brood at the beginning seemed to have a clearer start in brood rearing compared to the other colonies. However, in general not all colonies reared brood during the experiment, and some colonies started rearing brood but then stopped (Appendix, Figures A2 and A3).

**FIGURE 4:**
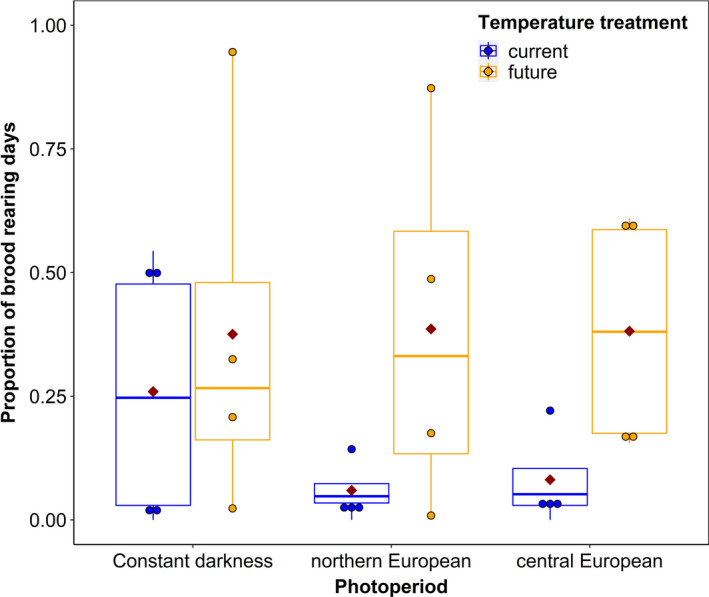
Proportion of days during which *Apis mellifera* colonies reared brood in the two temperature and three photoperiod treatments. Box plots display the median (thick bar), lower and upper quartile (boxes), and minimum and maximum values (whiskers) of the data set. The red diamonds represent the mean value of the data set. *n* = 4 colonies per unique combination of temperature and light treatment

Nevertheless, when we looked at the proportion of days during which colonies were rearing brood, we found that the temperature regime influenced the proportion of brood rearing days in colonies (*F* = 4.7, *p* = .05). Neither the photoperiod, the interaction of photoperiod and temperature, the origin of the colonies, or brood rearing at the beginning influenced the proportion of brood rearing days (photoperiod treatment: *F* = 0.35, *p* = .71; interaction: *F* = 0.9, *p* = .44; origin: 0.23, *p* = .64, brood at the beginning: *F* = 0.05, *p* = .83). In general under the future temperature regime, the colonies showed higher proportions of brood rearing days than in the current temperature regime (Figure 4).

### 3.3 Timing of flowering phenology and honey bee brood onset

Both photoperiod and species identity (*C. sieberi* vs. *A. mellifera*), as well as the interactions between the two factors, had a significant effect on the respective relevant phenological event, that is, timing of flowering or brood onset in the current and future temperature regime (Table 3).

**TABLE 3:** Results of mixed effects Cox models to test the difference between the flowering timing of *C. sieberi* and brood rearing activity timing of *A. mellifera* under the same temperature treatment (CT: Current temperature; FT: Future temperature)

Predictors	*C. sieberi* and *A. mellifera* starting week in CT			*C. sieberi* and *A. mellifera* starting week in FT		
	*χ* ^2^	*df*	*p* (>Chi)	*χ* ^2^	*df*	*p* (>Chi)
Photoperiod	8.9	2	.**01**	22.3	2	**<.0001**
Species	11.7	1	.**0006**	5.2	1	.**02**
Photoperiod × Species	11.1	2	.**004**	7.9	2	.**02**
Random effect (origin)						
Variance	0.00008			0.36		
Standard deviation	0.009			0.60		

Note

Bold values indicate significant differences between treatments (*p* < .05) according analysis of deviance based on chi‐square likelihood ratio tests.

**FIGURE 5:**
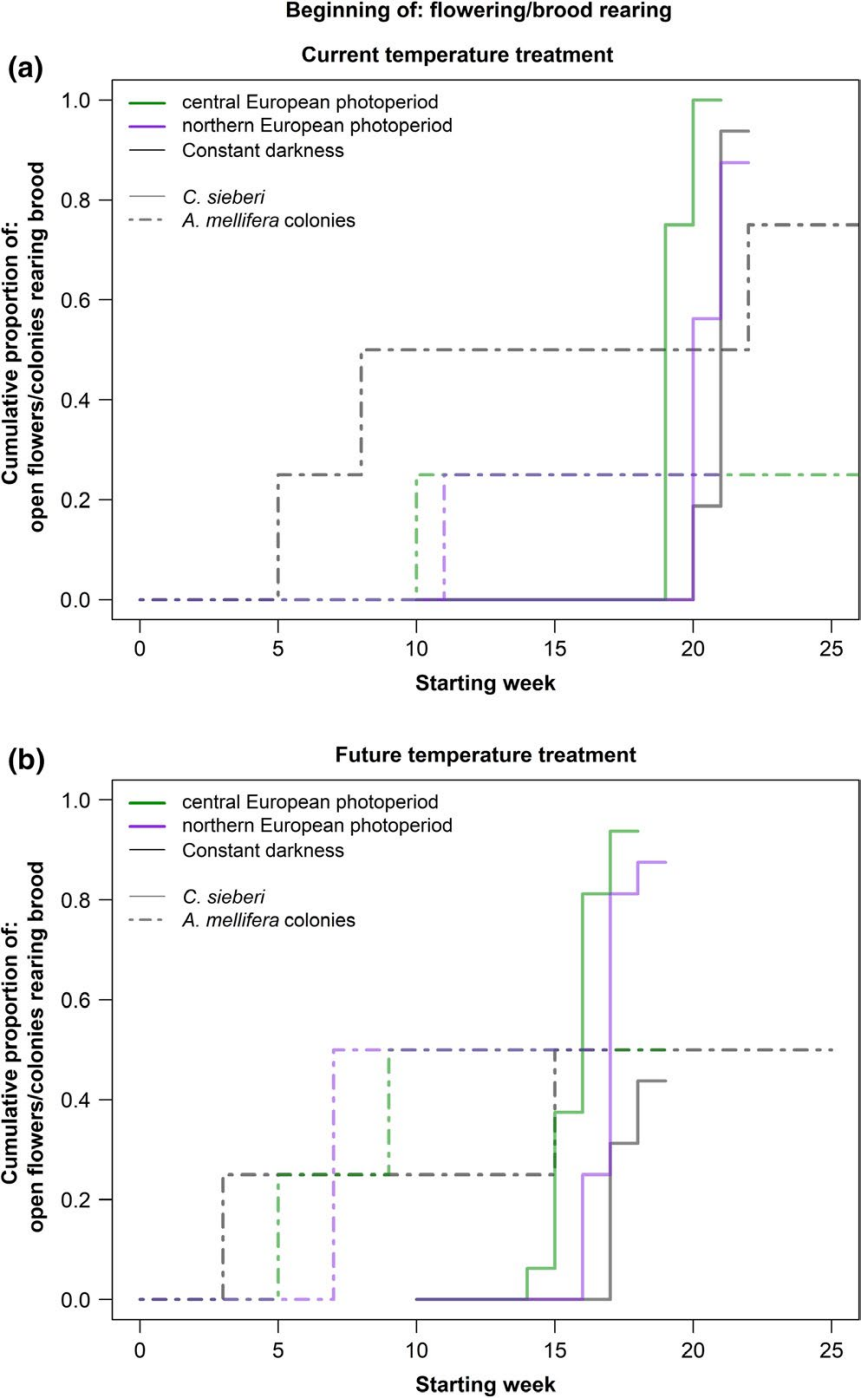
Timing of Flowering and brood rearing activity. (a) Cumulative proportion of open flowers of *Crocus sieberi* and *Apis mellifera* colonies rearing brood depending on the week of the experiment under the current temperature treatment and the different photoperiod regimes. (b) Cumulative proportion of open flowers of *C. sieberi* and *A. mellifera* colonies rearing brood depending on the week of the experiment under the future temperature treatment and the different photoperiod regimes

Specifically, the post hoc testing showed that under the current temperature and central European scenario, *A. mellifera* colonies started to rear brood significantly before than *C. sieberi* started flowering (Appendix, Table A2), approximately 9 weeks before (Figure 5, Appendix, Table A3). In the future temperature regime, only under constant darkness *A. mellifera* colonies started to rear brood significantly before than *C. sieberi* (Appendix, Table A2), also approximately 9 weeks before (Figure 5, Appendix Table A3). Under the other photoperiods and the two temperature treatments, we did not find a significant difference between *C. sieberi* and *A. mellifera* (Appendix, Table A2).

### 3.4 Weight change of the colonies

We also tested if our temperature treatments and the reproductive status of the colonies (rearing or not rearing brood) influenced the average weight change of the colonies. We found that when the colonies were rearing brood they had a larger daily weight loss (average weight change with brood: −28.84 g/day ± 2.91 g/day) than when they were not rearing brood (average weight change without brood: −20.15 g/day ± 0.91 g/day) (*F* = 7.1, *p* = .01). We did not find a significant effect of the temperature treatments, or the interaction between temperature treatment and the reproductive status, nor the origin of the colonies on the weight change (temperature treatment: *F* = 0.74, *p* = .4; interaction: *F* = 3.3, *p* = .08; origin: *F* = 0.02, *p* = .9).

## 4 DISCUSSION

In this study, we experimentally simulated overwintering conditions under current and future climate scenarios to explore the roles of temperature, photoperiod, and the dissociation between temperature and photoperiod as Zeitgebers on the timing of the flowering of an early seasonal geophyte and the brood rearing activity in Western honey bee colonies, a major generalist pollinator species.

As hypothesized, we observed an advance in the flowering phenology of *C. sieberi* growing under a future temperature regime, based on a predicted mean temperature increase of 4°C (IPCC, 2014), indicating that increased temperatures during winter and early spring can in fact advance its flowering phenology as has already been reported for other plant species (Kehrberger & Holzschuh, 2019; Menzel et al., 2006; Root et al., 2003). Moreover, *C. sieberi* growing under the photoperiod of the central European location with longer days in winter (Würzburg, Germany) flowered earlier than the ones under the northern European photoperiod with shorter days in winter (Snåsa, Norway). This indicates that in plants, photoperiod is used as a cue to indicate the change of the seasons (Vegis, 1964; Went, 1953), even though it has been reported that most geophytes species do not show a response to photoperiod (Khodorova & Boitel‐Conti, 2013).

We also noticed a difference in the final size of *C. sieberi* between the ones growing under the future temperature regime and the current temperature regime, with the plants under the future temperature regime being larger. This difference in size may result from the effect of temperature on cell elongation and hence growth (Went, 1953). Increased temperatures can also cause faster leaf senescence (Khodorova & Boitel‐Conti, 2013), which can shorten the time of resource availability for pollinators and herbivores. Interestingly, we only saw size differences between plants growing under the photoperiod of the northern and central European location in the current temperature regime but not in the future regime. These results might indicate that photoperiod fine‐tunes the processes behind growing and that higher future temperature can override such modulation which might reduce synchronization of flowering plants with suitable environmental conditions and pollinators.

Surprisingly, *C. sieberi* individuals which grew under constant darkness not only developed a flower bud but also flowered. This indicates that daily changes in temperature or the increase of temperature over time was sufficient to trigger flower development and opening. However, the lack of light did not allow these plants to obtain resources via photosynthesis, and therefore, not all of them had enough resources to develop flower buds and flower. The ability to flower under constant darkness has been reported for other plants (Chailakhyan, 1968; Lang, 1965).

It is long known, that plants have endogenous annual rhythms [i.e., rhythms that are present under constant environmental conditions (Aschoff, 1981)] that require external factors in order to be in synchrony with the environment (Bunning, 1956). The results of this study confirm that temperature and photoperiod are important environmental cues that also synchronize the internal rhythms of the geophytes with the seasons. Additionally, the fact that the increase in temperature caused a clear advance in the time of flowering, even when photoperiod indicated that winter would presumably continue, suggests that temperature was a critical factor that influenced the phenology of *C. sieberi*, while photoperiod fine‐tuned the timing of flower opening.

In general, *Apis mellifera* colony brood rearing activity was highly variable and several colonies did not start to rear brood during the experimentation time. This might have obscured treatment effects on colony phenology. Still, our results show that temperature had an effect on the proportion of days during which the colonies reared brood during winter. The colonies that were under the future temperature regime, that is, increased mean air temperatures, reared brood during more days than the ones under the current temperature regime.

The finding that higher temperatures during winter induced brood rearing activity, suggests that warmer winters (e.g., associated with ongoing climate change (IPCC, 2014)) could change brood rearing in *Apis mellifera* colonies. This might have detrimental effects on food storage (when a mismatch between the phenology of honey bee colonies and flowering plants occurs) and on varroa mite pressure (Nürnberger et al., 2019).

When trying to predict if *Apis mellifera* colonies and spring flowering plants are going to be in synchrony in a warming world, it is also necessary to consider the way both interacting species react to the dissociation of photoperiod and temperature as environmental cues for tracking the seasons.

On the one hand, *C. sieberi* not only relies strongly on temperature but also on photoperiod to time the start of flowering. This can mean that, even if temperature increases during winter, photoperiod can be used as cue that prevents *C. sieberi* from flowering too early in the year. On the other hand, in our experiment temperature influenced the proportion of brood rearing days but neither temperature nor photoperiod influenced the onset of brood rearing of *A. mellifera*. However, with our results we cannot conclude that temperature and photoperiod do not play a role in colony phenology, as under our experimental conditions not all colonies had a clear start in brood rearing.

Nonetheless, considering that: (a) in our experiment higher temperature during winter increased brood rearing activity, (b) temperature seems to be more important than photoperiod for inducing or synchronizing the biological clock that triggers brood rearing activity in *A. mellifera* colonies (Nürnberger et al., 2018), and (c) *A. mellifera* worker's first appearance date has advanced due to warmer winters (Gordo & Sanz, 2005, 2006; Sparks et al., 2010), temperature seems to be an important environmental cue that can synchronize *A. mellifera* colonies during winter.

On the whole, with increasing mean temperatures in winter and spring *C. sieberi* and *A. mellifera* colonies are likely to change their phenology and start to flower or, respectively, rear brood earlier in the year. This advance in the phenological event (i.e., timing of flowering or brood onset) of plants and pollinators can be similar, but it will depend on how the species respond to photoperiod, as the flowering of *C. sieberi* and the brood onset of *A. mellifera* colonies occurred or not at similar weeks depending on the photoperiod.

It should be recalled that not all the plants might advance their flowering with increases in temperature. Some plants species need cold temperatures to break the dormancy process (Asse et al., 2018; Yu et al., 2010). These differences in phenology could potentially lead to mismatches between flowering plants and *A. mellifera*. This mismatch could still be buffered by the large variation in brood rearing activity among honey bee colonies (as we saw in our experiment). This variation could ensure the availability of this dominant pollinator species under a large range of climatic conditions. However, as mentioned before even a few days of desynchronization could still lead to fitness losses in *A. mellifera*, as reported for solitary bees (Schenk et al., 2018), depending on remaining resource storages within the colonies and presence of alternative flowering resources (Nürnberger et al., 2019).

## 5 CONCLUSIONS

This study analyzed whether differences in the importance of temperature and photoperiod as Zeitgebers could lead to different phenological responses of the early‐season flowering geophyte *Crocus sieberi* and *Apis mellifera* colonies under future climates. In our study, we show that predicted future warmer temperatures advanced the flowering phenology of *C. sieberi* and increased the brood rearing activity of *A. mellifera* colonies. Importantly, photoperiod modulated the flowering phenology of *C. sieberi* but not the brood rearing phenology and activity of *A. mellifera*. The results confirm that increases in temperature can induce changes in phenological responses and suggest that photoperiod can also play a critical role in these responses, with currently unknown consequences for real‐world ecosystems in a warming climate.

## CONFLICT OF INTEREST

The authors reported no potential conflict of interest.

## AUTHOR CONTRIBUTIONS

**Gemma N. Villagomez:** Conceptualization (supporting); formal analysis (equal); investigation (equal); writing‐original draft (lead); writing‐review & editing (lead). **Fabian Nürnberger:** Conceptualization (lead); formal analysis (equal); investigation (equal); writing‐original draft (supporting); writing‐review & editing (equal). **Fabrice Requier:** Formal analysis (equal); writing‐original draft (supporting); writing‐review & editing (equal). **Susanne Schiele:** Conceptualization (supporting); investigation (equal); writing‐review & editing (supporting). **Ingolf Steffan‐Dewenter:** Conceptualization (lead); formal analysis (equal); funding acquisition (lead); resources (lead); supervision (lead); writing‐original draft (supporting); writing‐review & editing (equal).

## Data Availability

Data and R script are available in: https://doi.org/10.6084/m9.figshare.14401928.v1.

## APPENDIX A

**TABLE A1:** Pairwise comparison on the effect of temperature and photoperiod treatments on *C. sieberi* (CD: constant darkness photoperiod, CEP: central European photoperiod, NEP: northern European photoperiod)

Temperature	Comparison	*C. sieberi* start flowering week			*C. sieberi* final height			
		Estimate	*z*‐ratio	*p*	Estimate	*df*	*t*‐ratio	*p*
Current	CD‐CEP	−2.5	−5.3	**<.0001**	NA	NA	NA	NA
	CD‐NEP	−1.0	−2.2	.23	NA	NA	NA	NA
	NEP‐CEP	−1.6	−3.5	.**007**	−2.6	21.1	−3.7	.**007**
Future	CD‐CEP	−2.5	−5.3	**<.0001**	NA	NA	NA	NA
	CD‐NEP	−1.0	−2.2	.23	NA	NA	NA	NA
	NEP‐CEP	−1.6	−3.5	.**007**	−0.6	21.5	−0.9	.811

Photoperiod	Comparison							
CD	Current‐Future	−1.9	−4.6	.**0001**	NA	NA	NA	NA
CEP	Current‐Future	−1.9	−4.6	.**0001**	−2.9	21.1	−4.3	.**002**
NEP	Current‐Future	−1.9	−4.6	.**0001**	−4.8	21.5	−7.1	**<.0001**

Note

The first comparison was based on a mixed‐effects Cox model performed to test the effects of the treatments on its flowering timing. This comparison was performed with emmeans based on a model without interaction between temperature and photoperiod. The second comparison was based on a linear mixed effects model to test the effect of the treatments on the final height of *C. sieberi* considering the model with the interaction. The pairwise comparison was also performed with emmeans. Bold values indicate significant differences between treatments (*p* < .05).

**TABLE A2:** Pairwise comparison of photoperiod treatments based on mixed‐effects Cox models performed to test the difference between the flowering timing of *C. sieberi* and brood rearing activity timing of *A. mellifera* under the same temperature treatment (CT: Current temperature; FT: Future temperature, CD: constant darkness photoperiod, CEP: central European photoperiod, NEP: northern European photoperiod)

Photoperiod	Comparison	*C. sieberi* and *A. mellifera* starting week in CT			*C. sieberi* and *A. mellifera* starting week in FT		
		Estimate	*z*‐ratio	*p*	Estimate	*z*‐ratio	*p*
CD	*A. mellifera* – *C. sieberi*	0.4	0.7	.98	4.5	4.1	.**0006**
CEP	*A. mellifera* – *C. sieberi*	3.4	1.1	.**02**	2.3	0.7	.98
NEP	*A. mellifera* – *C. sieberi*	1.5	−1.5	.7	0.7	2.1	.3

Note

The pairwise comparison was performed with emmeans considering the models with the interaction between species identity and photoperiod. Bold values indicate significant differences between treatments (*p* < .05).

**TABLE A3:** Average and standard deviation of start flowering week and start brood rearing week for the different temperature and photoperiod treatments (CD: constant darkness photoperiod, CEP: central European photoperiod, NEP: northern European photoperiod)

Temperature	Photoperiod	*C. sieberi* start flowering week			*A. mellifera* start brood rearing week			Difference in phenological event *between C. sieberi* and *A. mellifera*
		Num. plants	Average (week)	*SD*	Num. colonies	Average (week)	*SD*	Week difference under same temperature
Current	CD	15	20.80	0.41	3	11.67	9.07	9.13
	CEP	16	19.25	0.45	1	10.00	NA	9.25
	NEP	14	20.36	0.50	1	11.00	NA	9.36
Future	CD	7	17.29	0.49	2	9.00	8.49	8.29
	CEP	15	15.80	0.86	2	7.00	0.00	8.80
	NEP	14	16.79	0.58	2	7.00	2.83	9.79

Photoperiod	Temperature	Number of advanced weeks due to the temperature treatment	
		Week difference under the same photoperiod	
		*C. sieberi*	*A. mellifera*
CD	Current‐Future	3.51	2.67
CEP	Current‐Future	3.45	3.00
NEP	Current‐Future	3.57	4.00

Note

Difference between *C. sieberi* and *A. mellifera* in the start of the phenological event and average advanced weeks due to temperature treatment.
